# The many faces of pediatric urticaria

**DOI:** 10.3389/falgy.2023.1267663

**Published:** 2023-11-03

**Authors:** Bulent Enis Sekerel, Deniz Ilgun Gurel, Umit Murat Sahiner, Ozge Soyer, Emek Kocaturk

**Affiliations:** ^1^ Department of Pediatric Allergy, Hacettepe University School of Medicine, Ankara, Türkiye; ^2^ Department of Allergology, Charite University School of Medicine, Berlin, Germany

**Keywords:** childhood, differential diagnosis, mimickers, urticaria, pediatric

## Abstract

Urticaria is a common disease that can affect individuals of all age groups, with approximately one-quarter of the population experiencing it at least once in their lifetime. Lesions characterized by erythema and itchy hives can appear anywhere on the body. These can vary in size ranging from millimeters to centimeters, and typically clear within 24 h. About 40% of patients with urticaria have accompanying angioedema, which involves localized deep tissue swelling. Urticaria usually occurs spontaneously and is classified into acute and chronic forms, with the latter referring to a condition that lasts for more than 6 weeks. The prevalence of chronic urticaria in the general population ranges from 0.5% to 5%, and it can either be inducible or spontaneous. The most common form of pediatric urticaria is acute and is usually self-limiting. However, a broad differential diagnosis should be considered in children with urticaria, particularly if they also have accompanying systemic complaints. Differential diagnoses of pediatric urticaria include chronic spontaneous urticaria, chronic inducible urticaria, serum sickness-like reaction, urticarial vasculitis, and mast cell disorders. Conditions that can mimic urticaria, including but not limited to cryopyrinopathies, hyper IgD syndrome, Periodic Fever, Aphthous Stomatitis, Pharyngitis and Adenitis (PFAPA), Tumor Necrosis Factor Receptor Associated Periodic Syndrome (TRAPs), and Schnitzler syndrome should also be considered. The many faces of pediatric urticaria can be both easy and confusing. A pragmatic approach relies on clinical foresight and understanding the various forms of urticaria and their potential mimickers. This approach can pave the way for an accurate and optimized diagnostic approach in children with urticaria.

## 1. Introduction

Urticaria, commonly known as hives, typically presents as a sharply circumscribed, superficial central swelling of varying sizes and surrounding erythema and is characterized by the skin returning to its normal appearance within 30 min to 24 h. The lesions can vary in size from millimetric to centimeters. Angioedema, a localized deep-tissue swelling, is associated with urticaria in 40% of patients and resolves within hours to days. Urticaria and angioedema are usually accompanied by an itching and burning sensation, respectively (1, 2).

**Figure 1:**
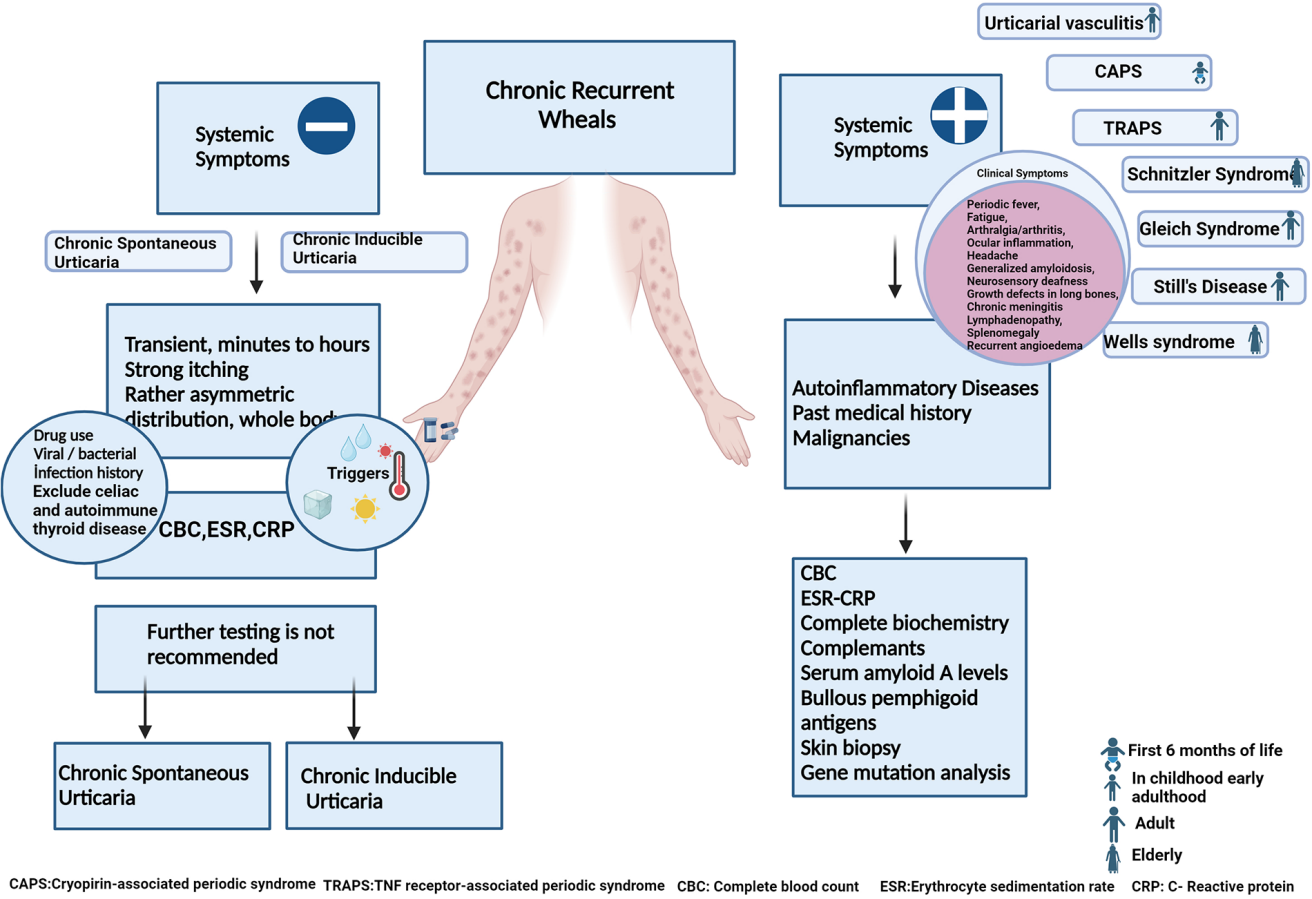
Differential diagnosis of childhood chronic urticaria. (Early adulthood: second and third decades of life).

In children, acute urticaria is the most commonly observed form of the condition, although chronic spontaneous urticaria (CSU) and chronic inducible urticaria (CındU) are also frequently seen, with a prevalence comparable to that in adults (1). While diagnosing urticaria is typically straightforward, healthcare providers must carefully consider other diseases and conditions that mimic urticaria. This consideration is crucial for ensuring an accurate diagnosis and appropriate management for children with urticaria (1, 2). Certain clinical clues can help differentiate urticaria from its mimickers. It is important to inquire about the spontaneous or inducible nature of the urticaria, its duration (acute or chronic), presence of systemic symptoms, characteristics of the lesions (urticaria or urticarial rash), traces upon healing, duration of more than 24 h, and accompanying symptoms such as itching, burning, pain, or angioedema. These questions often provide valuable information that greatly facilitates reaching a diagnosis (3, 4) (Table 1) (Figure 1).

**Table 1:** Differential diagnosis of urticaria.

Conditions presenting with urticaria
• Anaphylaxis • Exercise-induced anaphylaxis • Acute urticaria • Chronic urticaria - Chronic spontaneous urticaria - Chronic inducible urticaria • Contact Urticaria/Urticarial Contact Dermatitis • Mast cell activation syndrome
Conditions presenting with urticarial lesions
• Serum sickness-like reaction • Kawasaki disease • Papular urticaria • Urticaria Multiforme • Juvenile idiopathic arthritis • Mastocytosis • Urticarial vasculitis • Autoinflammatory urticarial syndromes - Cryopyrin associated periodic syndrome (CAPS) - TNF receptor-associated periodic syndrome (TRAPS) - Mevalonate kinase deficiency (Hyper IgD syndrome) - Periodic fever, aphthous stomatitis, pharyngitis, and adenitis syndrome (PFAPA) - Schnitzler syndrome - Adult Onset Still’s Disease (AOSD) • Wells syndrome and hyper-eosinophilic syndrome • Bullous pemphigoid • Frey syndrome

Urticaria is a prevalent and time-consuming condition encountered in pediatric practice. While it is generally diagnosed and managed promptly, atypical presentations and clinical courses can pose challenges. The primary objective of this review article was to provide a pragmatic approach and comprehensive summary of urticaria and its mimickers, thereby aiding clinicians in accurate diagnosis and effective management.

## 2. Conditions presenting with urticaria

### 2.1. Anaphylaxis

Anaphylaxis is a severe and potentially life-threatening reaction that usually occurs within seconds or minutes of exposure to an allergen. Several distinguishing characteristics can aid in the differentiation between anaphylaxis and urticaria. Anaphylaxis typically manifests with a sudden onset, occurring within minutes to hours after exposure to an allergen, whereas urticaria tends to develop more gradually and can persist for longer. Anaphylaxis often co-exists with urticaria, but is characterized by the presence of additional systemic symptoms. These symptoms can affect various organ systems, including the gastrointestinal, respiratory, cardiovascular, and neurological systems. Urticaria typically resolves within a few hours to several days, whereas anaphylaxis can have a shorter duration but may persist for longer if not promptly treated (5).

Anaphylaxis affects multiple systems in the body and can progress rapidly if left untreated. The signs and symptoms of anaphylaxis can vary, but mostly (>80%) include skin reactions, such as urticaria, angioedema, pruritis, flushing, or pallor. Gastrointestinal symptoms include nausea, vomiting, abdominal pain, or diarrhea. Morbidity and mortality are high if there is respiratory or cardiovascular system involvement (6). Respiratory symptoms include dyspnea, wheezing, stridor, cough, and chest or throat tightness. Cardiovascular symptoms are characterized by tachycardia, hypotension, light-headedness, dizziness, or fainting (7). A feeling of impending doom or anxiety may also accompany these symptoms. In childhood, anaphylaxis is most commonly triggered by food allergens such as cows'milk, hens’ eggs, peanuts, tree nuts, sesame, and shellfish, followed by medications (e.g., penicillin, NSAID), and insect stings (e.g., honey bees, wasps), or latex. Prompt treatment is critical for the prevention of mortality, and the primary treatment involves the administration of intramuscular epinephrine (adrenaline) (6, 7).

### 2.2. Exercise-induced anaphylaxis

Exercise-induced anaphylaxis is characterized by the development of anaphylaxis, including hives, dyspnea, hypotension, and systemic reactions, triggered explicitly by physical activity or exercise. Many patients notice a tingling, itching, or burning sensation on the skin 10–25 min after exercise before hives appear. The macules may coalesce to form large areas of erythema (8). It is considered a rare but potentially severe condition. The exact cause of exercise-induced anaphylaxis is not fully understood but is believed to involve a combination of factors, including physical exertion, specific foods or medications consumed before exercise, and sometimes other environmental factors (9).

In exercise-induced anaphylaxis, the appearance of urticaria usually coincides with or shortly follows the onset of physical activity. Urticarial lesions are often widespread, affecting various areas of the body. They can occur on the skin surface exposed to friction during exercise, such as the thighs, trunk, arms, and neck. Urticaria in exercise-induced anaphylaxis is accompanied by intense itching, which may be a notable symptom during physical activity. The individual hives are typically larger in size (often >1 cm in diameter) and may coalesce to form larger areas of swollen and red skin. The hives may persist for variable durations, ranging from minutes to hours after the exercise has ceased (10).

It is important to consider the sudden onset of other systemic symptoms, such as respiratory distress, gastrointestinal symptoms, and cardiovascular changes, as these can help differentiate this condition from isolated urticaria.

**Figure 2:**
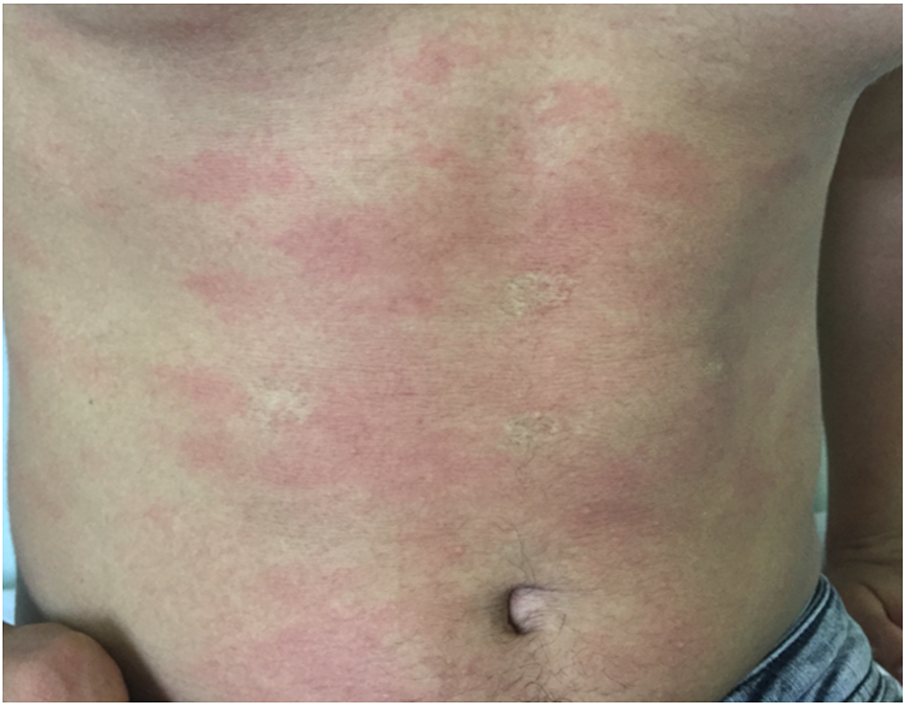
A 17-year-old male with cholinergic urticaria with minute urticarial papules and a pronounced flare reaction.

In some cases, exercise alone can trigger anaphylaxis, whereas in others, the combination of exercise and specific triggers, such as certain foods or medications, is necessary. Food-dependent exercise-induced anaphylaxis (FDEIA) is an exercise-related allergy after food intake. The most commonly reported triggering foods are wheat, celery, grapes, nuts, peaches, eggs, rice, tomatoes, seafood, and cheese (11). Cholinergic urticaria (CholU), FDEIA, and heat urticaria can be differentiated according to whether this condition develops secondary to exercise and/or heat stimulation (12). CholU is another entity characterized by small 1–3 mm punctate papules, usually surrounded by an erythematous halo (Figure 2). It typically starts on the upper chest and neck but can spread over the whole body. A single lesion typically resolves within 15 to 20 min. Exercise, emotional stress, and a sudden increase in body temperature are frequent etiologies (13). There appears to be an increase in plasma histamine levels in patients with symptoms. As the exposure to triggering factor continues, hives may coalesce and resemble angioedema, but vascular collapse is rare (13).

### 2.3. Acute urticaria

**Figure 3:**
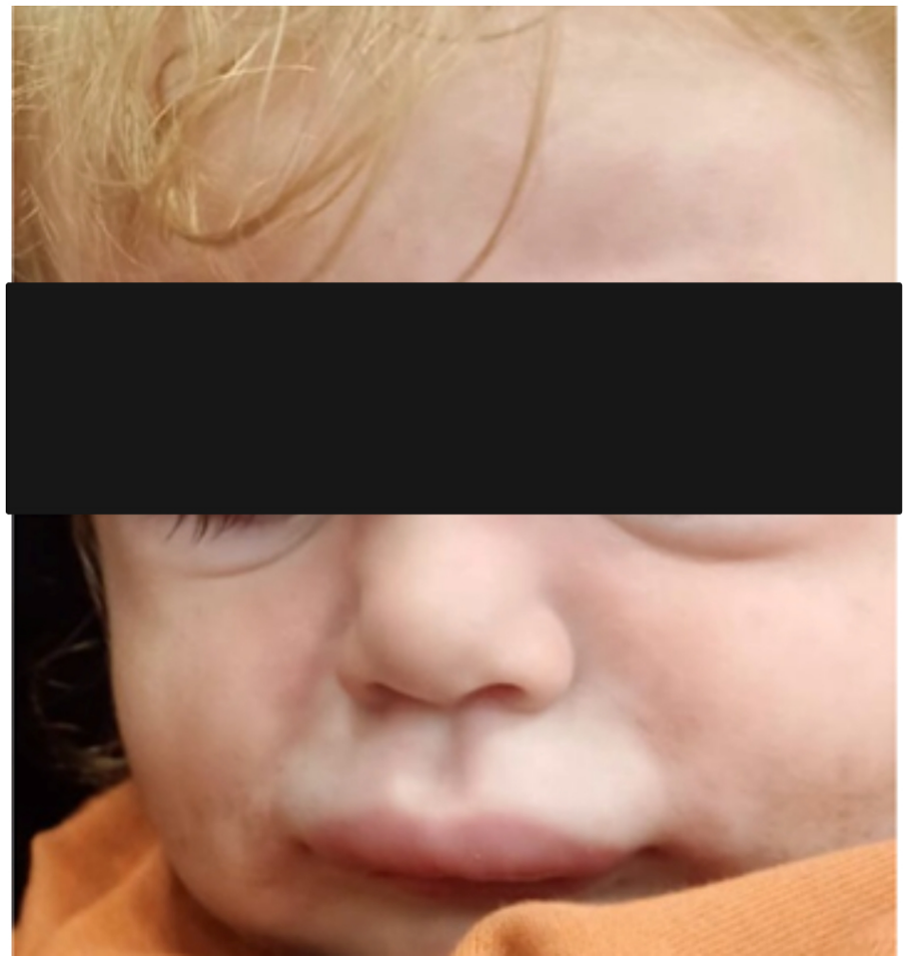
Acute urticaria with angioedema in a child.

Urticaria is the most prevalent form of pediatric urticaria, affecting up to 25% of the pediatric population (14). It can manifest either sporadically, with individual episodes lasting from a few hours to several days, or it may recur in response to various triggers. While it can affect children of all ages, it is more frequently observed in younger children. Interestingly, there is no significant gender predominance, as it affects both boys and girls equally. Approximately 40% of cases of acute urticaria may present with concomitant angioedema (15) (Figure 3). Infections, such as viral and bacterial infections, are the most common triggers (48%) for acute urticaria (4, 15). For example, urticaria accounts for approximately 10%–20% of cutaneous manifestations in pediatric patients with COVID-19 (16, 17). Although infectious causes primarily trigger acute urticaria, it is not uncommon to blame antibiotics used in treating infection for its etiology (15).

A study evaluating urticaria ICD codes suggested that the most common causes of urticaria after infection are drugs, foods, insect bites, pollen, blood products, and vaccination (18). The rates of urticaria cases attributed to food and drug hypersensitivity are 2.7% and 5.4%, respectively, among all cases of acute urticaria (15).

### 2.4 Chronic urticaria

(Table 2).

**Table 2:** Characteristics to distinguish chronic urticaria and differential diagnosis.

Diseases	Skin	Duration of lesions	Localization	Other dermatological symptoms	Occurrence of angioedema	Systemic symptoms	Characteristic features
Chronic Spontaneous Urticaria	Wheals and/or swellings	Hour to 24 h	Asymmetric distribution pattern throughout the body	Pruritus	Possible (∼ 40%–50%)	Rare	Good response to H1 antihistamines or omalizumab
Urticarial Vasculitis	Wheals with/without swellings	>24 h in most cases	Whole body	-Pruritus	Can be seen	Gastrointestinal,pulmonary and renal involvement	Fades leaving a trace /postinflammatory hyperpigmentation, histology with leukocytoclastic vasculitis
				-Burning, pain			
				-Fades leaving a trace			
						joint and muscle pain	
						Fever	Response to immunosuppressive medication
Cryopyrin Associated Periodic Syndrome	Cold-dependent appearance of wheals, maculopapular exanthema	Hour to 24 h	Rather symmetrical, face less often affected.	Burning	Not described	Fever, weakness, arthralgia, arthritis, ocular inflammation, headache generalized amyloidosis, neurosensory deafness.	Cold-related complaints
							(Pruritus post cold exposure)
- FCAS - Muckle-Wells syndrome - NOMID							Good response to anti IL-1 treatment
							Ice cube test is negative.
							While fever is not seen with FCAS
						Growth defects in long bones chronic meningitis	
PFAPA	Atypical	Recurrence every	Trunk and extremities	-	-	High fever	Periodic fever
	Rashes					- Pharygngitis - Cervical - Adenitis	
		3–8 weeks					
						Stomatitis	
Schnitzler Syndrome	Wheals	Hours to 24 h	Rather symmetrical, often on the trunk of the body	Burning	Very rare	Fever, arthralgias, muscle, bone pain, fatigue	Monoclonal gammopathy
							Good response to high dose steroids
Adult Onset Still's Disease	Erythematous, macular eruption	7–10 days	Usually affects the trunk	Pruritus	Very rare	High fever, arthralgia, Lymphadenopathy, splenomegaly	LACC1 gene
				Cutaneous pain			HLA DRB1
			Whole body				
	Rash						
	Bright, salmon-pink, morbilliform						

#### 2.4.1. Chronic spontaneous urticaria

**Figure 4:**
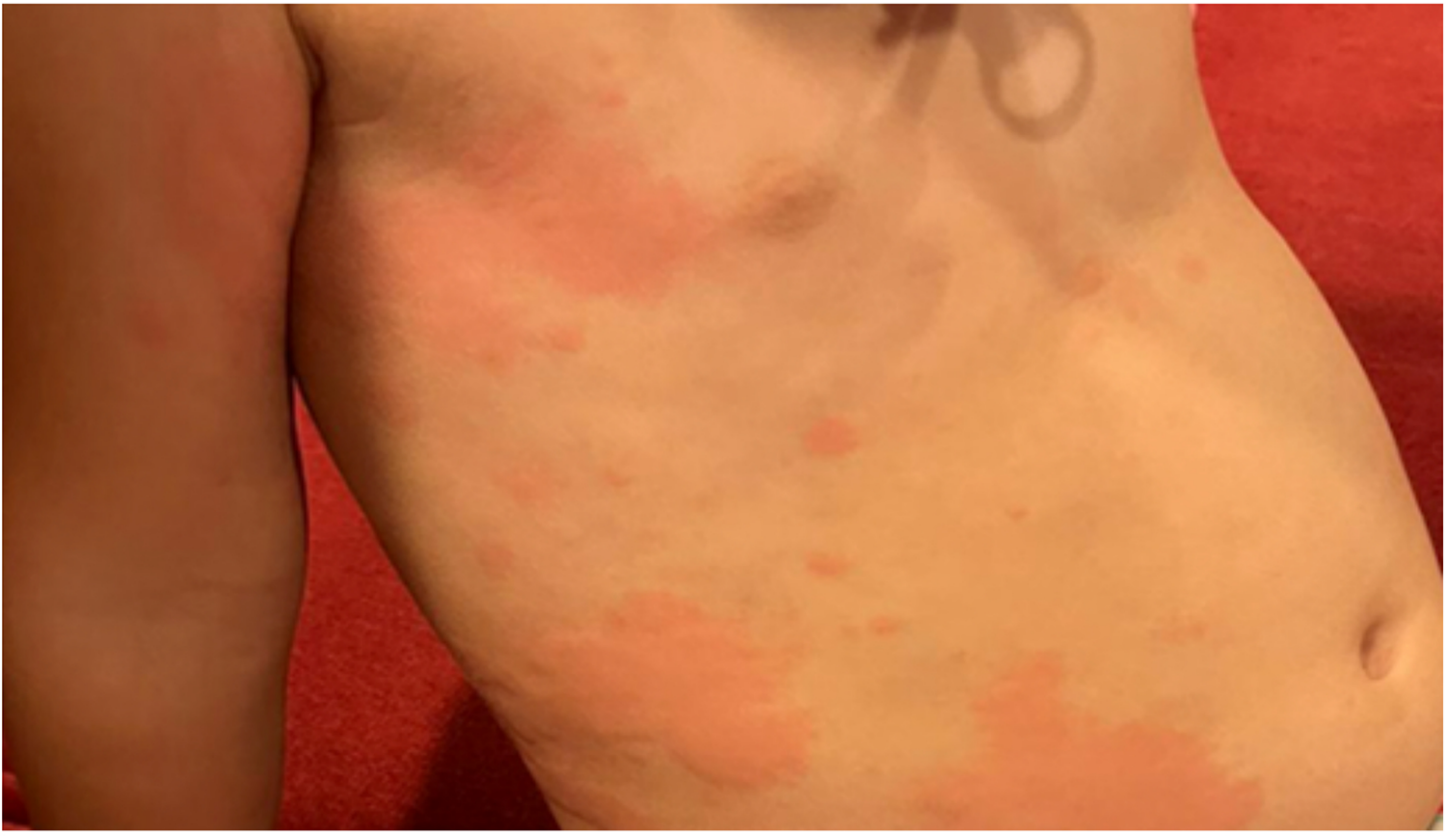
Wheals on the body of a child with chronic spontaneous urticaria.

Chronic spontaneous urticaria, previously named chronic idiopathic urticaria, is a long-lasting form characterized by hives/wheals that occur spontaneously without an identifiable trigger (1). Urticaria is considered chronic when it persists for more than six weeks, and in up to 40% of cases, chronic urticaria is accompanied by episodes of angioedema. The two conditions share similar underlying mechanisms, triggers, and treatment approaches. Wheals almost always resolve spontaneously in less than 24 h without a trace, while angioedema may take several days (1) (Figure 4).

Several factors are known to be associated with the development or exacerbation of chronic urticaria including autoimmune factors, physical stimuli (such as pressure, friction, heat, cold, sunlight, or exercise), infections, drugs such as non-steroidal anti-inflammatory drugs (NSAIDs), antibiotics (e.g., penicillin), and antihypertensives (e.g., ACE inhibitors), food and food additives (rare, such as preservatives or colorings), allergens (some individuals may experience hives as a result of exposure of specific allergens) and emotional stress. CSU also appears to be one of the leading risk factors for hypersensitivity to NSAIDs in childhood (1, 19, 20).

The prevalence of pediatric CSU is 0.8%, which is a comparable rate to that of adults (21). CSU does not show significant gender predominance and affects both boys and girls equally (22, 23).

In about half of cases, CSU may resolve within 5 years, but recurrence is common (19, 24, 25). The intermittent nature of the condition can have a significant impact on a child's quality of life, leading to itching, discomfort, and potential psychological effects (1, 3). Consequently, early diagnosis, appropriate management, and regular follow-ups with a healthcare provider are imperative to provide relief and enhance the overall well-being of affected children. Although antihistamines are the first-line treatment of CSU, complete response can only be achieved in children with low disease activity (26, 27). Addressing these aspects comprehensively can lead to better outcomes and improved quality of life for children living with CSU.

Chronic spontaneous urticaria is thought to involve an abnormal immune response, although the exact underlying mechanisms are not fully understood (28). It may be associated with autoimmunity/autoallergy, where the immune system mistakenly targets and activates mast cells and basophils, leading to the release of histamine and other inflammatory mediators (25, 29, 30).

#### 2.4.2. Chronic inducible urticaria

**Figure 5:**
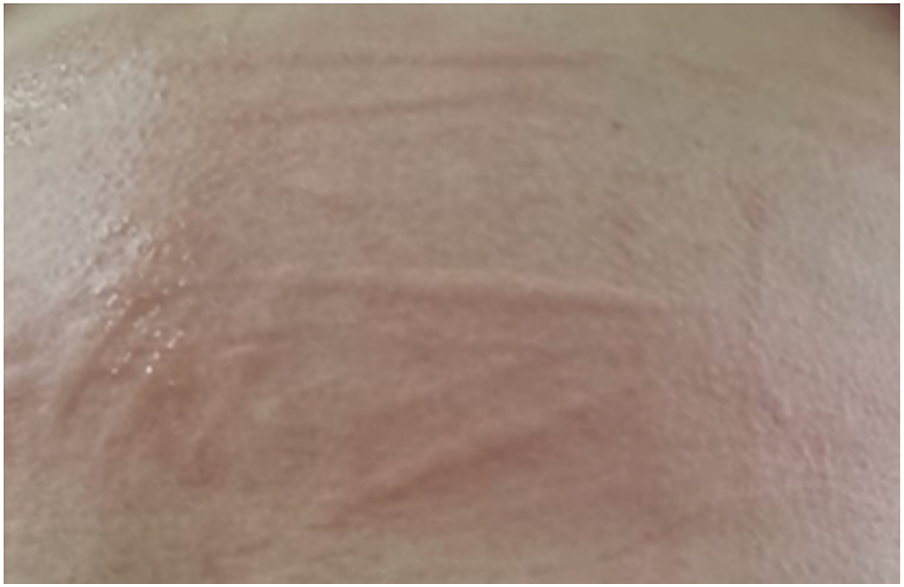
Symptomatic dermographism on the back of a child with linear wheals appearing after scratching the skin.

Chronic inducible urticaria, previously known as physical urticaria, is a form of chronic urticaria where certain stimuli specifically trigger hives (Figure 5). Unlike CSU, CIndU is characterized by hives induced by specific stimuli (1). A straightforward history facilitates diagnosis. Confirmatory tests are defined for cold, heat, cholinergic, dermatographism, sun, vibration, and water-induced urticaria subtypes (Table 3). It is usually transient, lasting only a few hours, and shows no cellular infiltration in skin biopsies. Only delayed pressure urticaria lesions are an exception type of CIndU, which can last for approximately 24 h and have cellular infiltration (30).

**Table 3:** Classification of chronic inducible urticaria and the diagnostic approach.

Subtypes	Diagnosis	Diagnostic test	Treatment
Solar Urticaria	History-Provocation test	UV-A/UV-B → 6 J/cm^2^–60 mJ/cm^2^.	Second Generation H1 Antihistamines
		In children with a negative response, sensitivity to light can be tested from a distance of 10 cm. Causes a rapid urticarial response within 10 min. (31)	Anti IgE treatment (32)
			Intravenous
			Immunglobulin (33)
Cholinergic Urticaria	History- Provocation test	Exercise should be performed until sweating and up to 15 min after the onset of symptoms. The test is positive if exercise produces typical redness for 10 min (34)	Second Generation H1 Antihistamines
Aquagenic Urticaria	History- Provocation test	Water-borne urticaria, a towel moistened with 36°C water or saline is placed on the body of the patient. Positive response if urticarial lesions develop at the contact site within 10 min after removing the compress/towel (35, 36)	No special recommendations
Symptomatic Dermographism	History- Dermagrophometer	The object should be held plumb line to the skin and smooth pressure should be applied to the volar forearm or upper back. It should not be inflamed. A positive response if an itchy, palpable bump is present within 10 min of the provocation.	Second Generation H1 Antihistamines (37)
			Omalizumab (38)
		FricTest	
Cold Urticaria	History- TempTest	The ice cube.	Cetirizine
		TempTest (39)	Ketotifen (40)
			Loratadine (41)
			Hydroxyzine (42)
			Rupatadine (43)
Hot Urticaria	History- Provocation test	Heat provocation test methods (44) - Hot water baths, - TempTest	Cetirizine
			Fexofenadine
Late Pressure Urticaria	History- Provocation test	- 7 kg shoulder strap - Dermographometer	Loratadine + montelukast (45)
Contact Urticaria	History -Exogenous Agent	Urticaria occurs after contact with the suspected agent by immunological and non-immunological mechanisms. The test consists of gently dabbing 0.1 ml of the test substance over an area of intact skin on the volar side of the forearm. The best tool: - an alcohol-water mixture - alcohol-propylene glycol mixture.	No special recommendations

Managing CIndU involves identifying and avoiding specific triggers whenever possible. Antihistamines are typically the first-line treatment to help alleviate symptoms and reduce the frequency and severity of hives (31, 46).

### 2.5. Contact urticaria/urticarial contact dermatitis

Contact urticaria and urticarial contact dermatitis are two distinct skin conditions, which although sharing some similarities, exhibit differences in their characteristics.

Contact urticaria typically manifests as the development of hives and itching at the point of contact with an allergenic or irritating substance. These symptoms arise swiftly, often within minutes of exposure to the triggering agent. Common culprits encompass foods, latex, specific chemicals, plants, and insect bites. The manifestations of contact urticaria typically subside within a few hours once the triggering substance is removed (47).

Urticarial Contact Dermatitis also presents with hives and itching at the site of contact with an allergen or irritant. The visual appearance of the rash resembles that of contact urticaria. However, in urticarial contact dermatitis, the rash emerges relatively rapidly, akin to contact urticaria, but can also occur several hours to a day following exposure. Urticarial contact dermatitis stems from allergic or irritant reactions upon skin contact with particular substances, including certain chemicals, plants (such as poison ivy or poison oak), or metals (e.g., nickel) (48, 49). Unlike contact urticaria, urticarial contact dermatitis can persist for days or even weeks, especially with continued exposure to the triggering agent.

In children, the most frequent causes of contact urticaria are foods, which are particularly prevalent in children with atopic dermatitis. Food-associated contact urticaria typically results from immunological reactions (50). Nevertheless, instances of non-immunological contact urticaria related to foods do exist, often linked to items such as tomatoes, ripe cheese, pineapple, red wine, contaminated tuna, and yeast (51). Furthermore, additives and preservatives, such as benzoic acid, sorbic acid, cinnamic acid, and cinamic aldehyde, incorporated into foods, can also induce contact urticaria through the same mechanisms.

### 2.6. Mast cell activation syndrome

Mast Cell Activation Syndrome (MCAS) is a condition characterized by the abnormal activation and release of mast cell mediators throughout the body. It is considered a subset of mastocytosis, but unlike mastocytosis, MCAS does not involve a significant increase in mast cell numbers (52). The symptoms of MCAS can vary widely and affect multiple organ systems. Common symptoms include skin and gastrointestinal manifestations. Skin symptoms include flushing, itching, urticaria, angioedema, and dermatographism. Gastrointestinal symptoms are abdominal colic, bloating, diarrhea, nausea, and vomiting. Respiratory symptoms (wheezing, dyspnea, nasal congestion, and throat tightness) and cardiovascular symptoms (tachycardia, dysrhythmia, hypotension, and fainting) may accompany them. Patients may experience idiopathic and allergic anaphylactic shock. Abnormalities in laboratory tests are common and varied. Biomarkers such as urinary histamine metabolites, tryptase, urinary PGD2 metabolites, and urinary leukotriene 4 metabolites can be used to diagnose the disease (53).

Unlike urticaria, MCAS has a wide range of systemic symptoms that go beyond just skin manifestations. In addition, the diagnosis of MCAS includes evaluation of the levels of mast cell mediators such as tryptase during both asymptomatic and symptomatic episodes (54).

## 3. Conditions presenting with urticarial lesions

### 3.1. Serum sickness-like reaction

Serum sickness-like reaction, also known as drug-induced SSLR, is a type III hypersensitivity reaction that can occur in response to certain medications. It resembles serum sickness, but the term “serum sickness-like” is used because it typically occurs without the exposure of serum or its components (55). In SSLR, the immune response leads to the formation of immune complexes, which deposit in various tissues. These immune complexes trigger an inflammatory response, causing skin rash, fever, fatigue/malaise, lymph node enlargement, and gastrointestinal symptoms (56).

**Figure 6:**
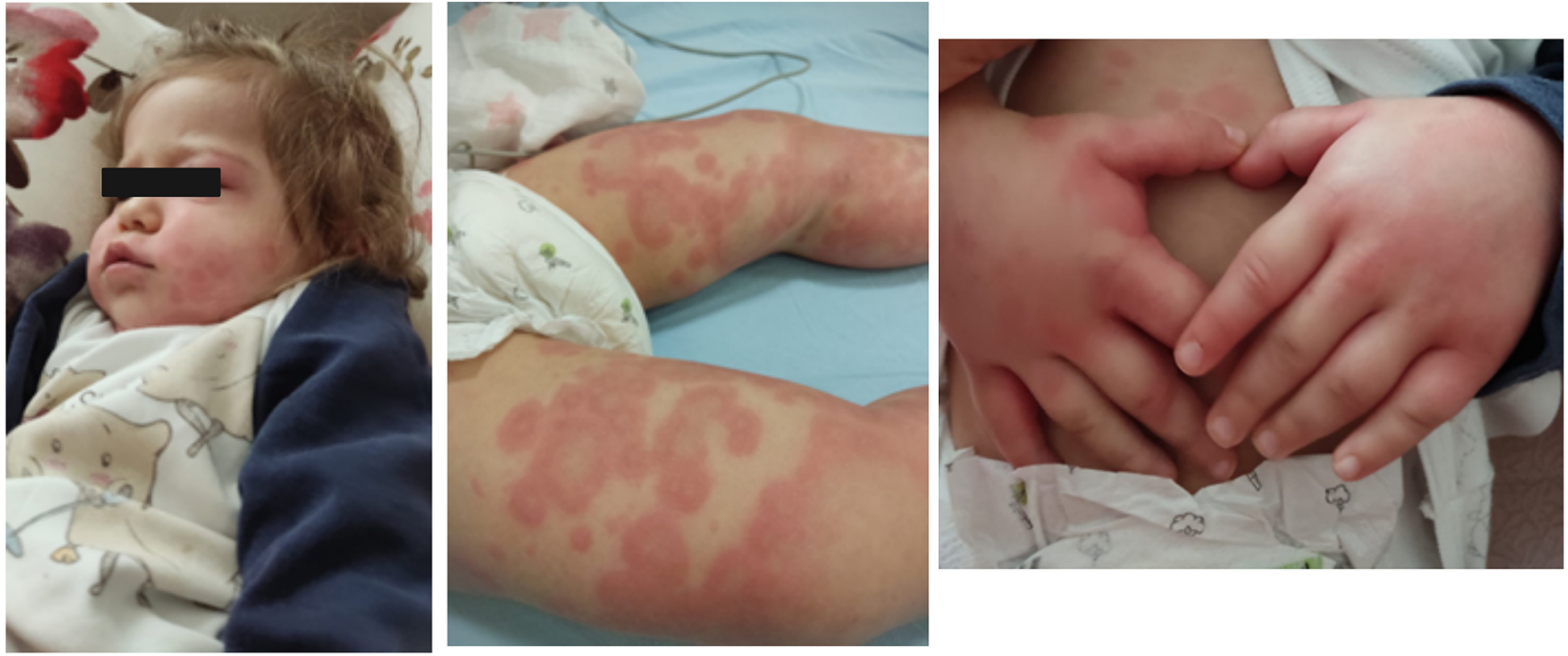
Amoxicillin induced serum sickness-like reaction: eyelid edema, urticarial plaques with purpuric appearance, and hand edema.

Serum sickness-like reaction can occur in a broad age range from infancy to adolescence. In the study of Magana et al. (55), which included 83 patients aged 11 months to 12 years, the mean time between exposure to the drug and the development of symptoms was recorded as 8 days in 80% of the patients with amoxicillin. Urticaria and similar rashes were present in 35% and erythema multiforme in 38.5% of the cases. Furthermore, hand and foot arthritis was present in 60% of the study cohort (55). Itching, lip/eye swelling, and fever have been reported in approximately 30%–45% of patients. Erythematous, annular, edematous, urticaria-like plaques evolving to ecchymotic patches may occur together or separately. Echymotic-colored erythematous urticarial plaques of variable size often appear one to three weeks after drug exposure with swelling of the hands and feet (Figure 6). It is frequently seen on the face, trunk, and extremities and has no mucosal involvement (57). It may be accompanied by fever, weakness, irritability, and difficulty in walking. Most patients recover without complications. Systemic corticosteroids and antihistamines facilitate recovery (57).

### 3.2. Kawasaki disease

Kawasaki disease primarily affects children under the age of five and is characterized by the involvement of medium-sized blood vessels, including the coronary arteries (58, 59). The exact cause of Kawasaki disease is unknown, but it is believed to involve an abnormal immune response, possibly triggered by an infection. The characteristic symptoms of Kawasaki disease include prolonged fever (lasting for at least five days), and rash (on the trunk, extremities, or genital area), which is often described as erythematous and may be accompanied by peeling of the skin, particularly on the hands and feet, red eyes, strawberry tongue, enlarged lymph nodes (particularly in the neck area) and edema on the hands and feet (59). Skin lesions include a diffuse, tender, erythematous plaque and eruptions with morbilliform, urticarial, and scarlatiniform morphologies that take several days to settle (60). Prompt diagnosis of Kawasaki disease is crucial as it can lead to complications such as coronary artery aneurysms or myocarditis. Treatment involves high-dose intravenous immunoglobulin (IVIG) and aspirin to reduce the risk of complications (61).

### 3.3. Papular urticaria

**Figure 7:**
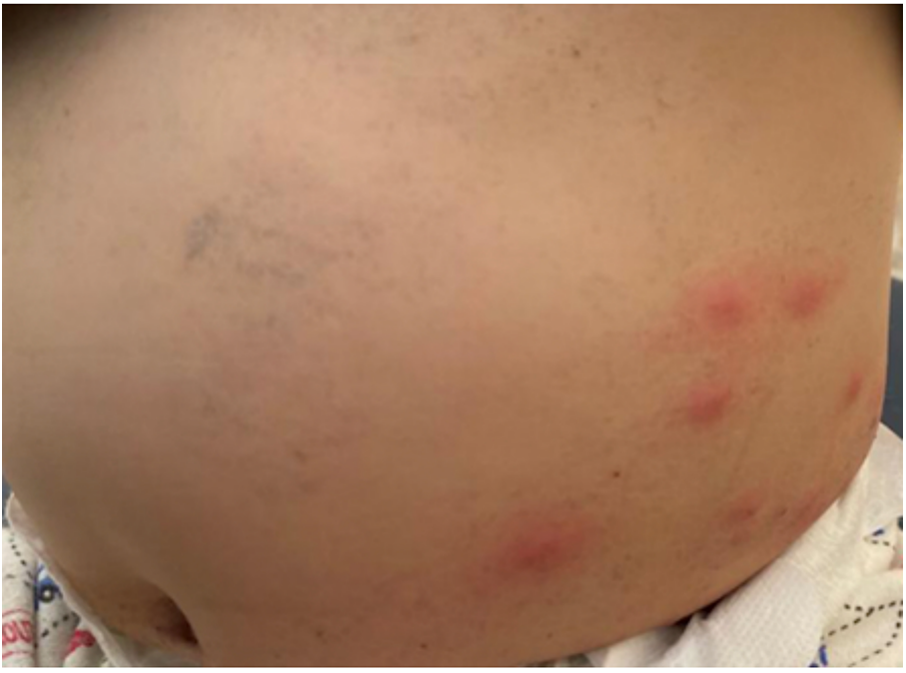
Papular urticaria (insect bite) in an infant after flea bites.

Papular urticaria results from a chronic, recurrent hypersensitivity reaction that often develops late after insect bites (62, 63). The lesions may look like urticaria, but urticaria lesions last shorter than 24 h and the lesions disappear without leaving a mark. Erythematous itchy papules, isolated or combined, sometimes with vesicles overlying, are apparent on mainly exposed areas such as the face and extremities (63) (Figure 7). Vesicle or itching at the bite site or a crust may be noticed at the site of the ruptured vesicle. The lesion reaches its maximum size in 2–3 days but may persist for longer and will heal spontaneously (64). No further laboratory examinations are required.

### 3.4. Urticaria multiforme

**Figure 8:**
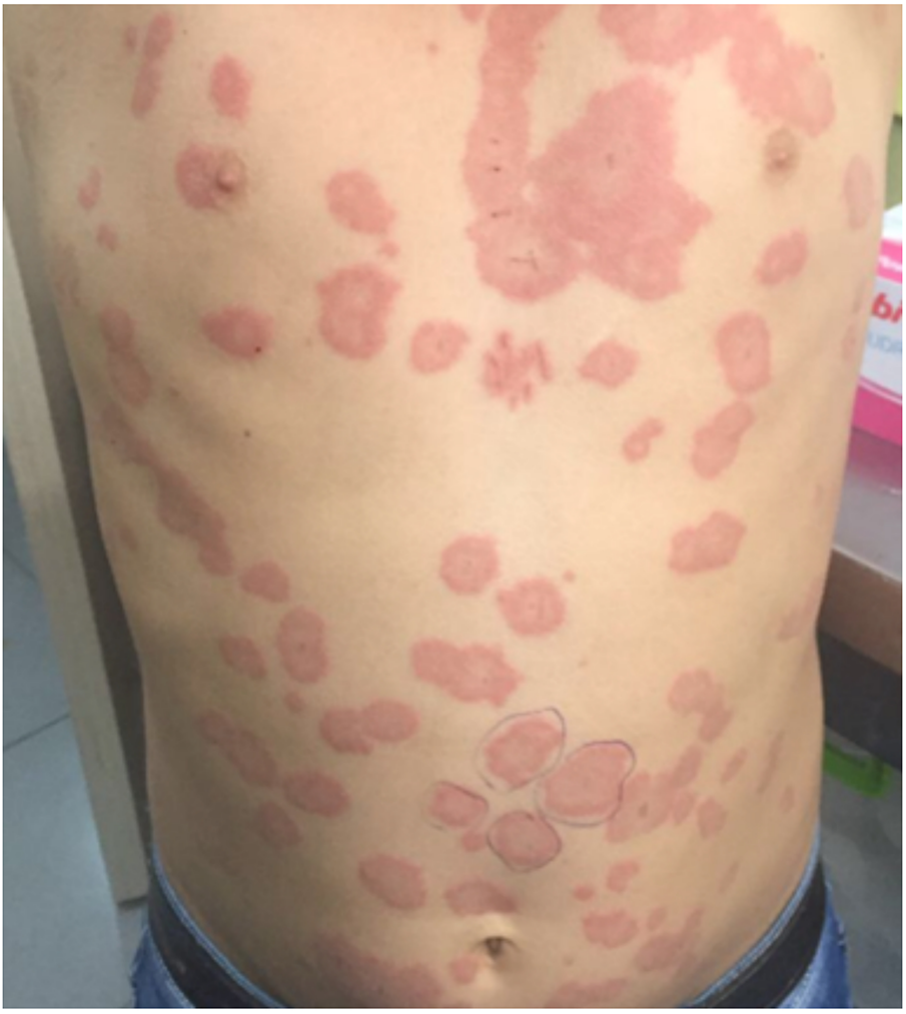
Urticaria multiforme in a 16-year-old male.

Urticaria multiforme, previously referred to as “acute annular urticaria” (65, 66), describes a cutaneous reaction that often follows a viral infection and shares morphological characteristics with acute urticaria. However, unlike typical urticaria, urticaria multiforme presents as polycyclic to annular urticarial lesions with bruise-like areas, resembling SSLR (55, 57). The central dusky appearance within an annular, erythematous, urticarial plaque may be mistaken for erythema multiforme (Figure 8). Urticaria multiforme is commonly observed in children between the ages of 4 months and 4 years, but can occur at any age. Preceding the development of the rash, symptoms may include a fever lasting 1–3 days, with/without other viral illness-related symptoms such as common cold, otitis media, and gastroenteritis (67).

Diagnostic criteria for urticaria multiforme include: (i) Annular to polycyclic urticarial lesions with transient ecchymotic skin changes, (ii) Individual lesions lasting less than 24 h, (iii) Presence of angioedema or acral edema, (iv) Dermatographism, (v) Increased levels of acute phase reactants, (vi) Favorable response to antihistamines (63). Unlike erythema multiforme, urticaria multiforme lacks true target lesions, skin necrosis, mucous membrane involvement, or blistering (63). Although it shares pathogenic similarities with urticaria, urticaria multiforme exhibits distinct morphological features. The occurrence of a hemorrhagic morphology within urticarial lesions in children is commonly observed, although the exact reasons for this remain unknown. In a prospective study on urticaria in children aged 1 to 36 months, the characteristic hemorrhagic pattern now recognized as urticaria multiforme was observed in nearly half of the cases (65, 66).

### 3.5. Juvenile idiopathic arthritis

Juvenile idiopathic arthritis (JIA) is an inflammatory disease that occurs before the age of 16 years, and is characterized by arthritis lasting at least 6 weeks. It is the most common form of arthritis in childhood (68).

Cutaneous involvements are non-fixed erythematous rashes. The rash associated with systemic-onset JIA typically appears early in the course of the disease, often before arthritis develops. It is usually one of the first noticeable signs, together with systemic symptoms such as fever, fatigue, and other general malaise. The rash in systemic-onset JIA is characterized by salmon-pink or light red spots, and it is often described as “evanescent” because the rash comes and goes, changing its location and appearance. The rash can be transient, with individual spots lasting for a few hours to a few days and then disappearing. New spots may appear while others fade away, leading to a migratory pattern (69).

Studies have shown that IL-6 plays a role in the pathogenesis of the disease, and therefore, tociluzumab, an anti-IL-6 receptor antibody, is beneficial in the treatment of the disease (70).

### 3.6. Mastocytosis

**Figure 9:**
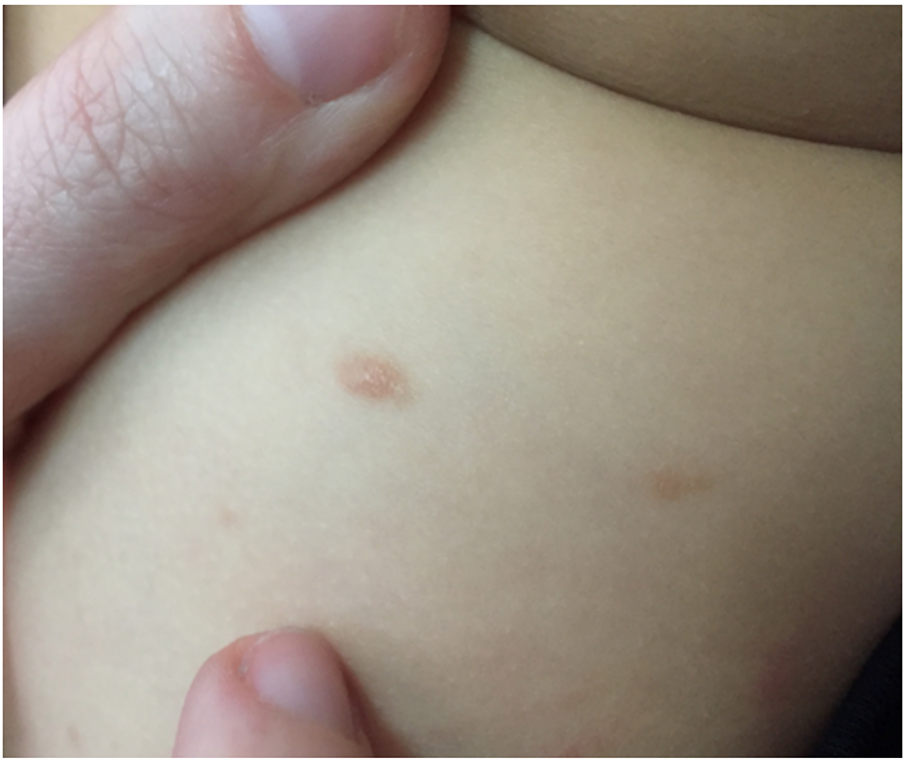
Cutaneous mastocytosis in a 4-year-old girl.

Mastocytosis is a rare disorder characterized by the abnormal accumulation and activation of mast cells in various tissues throughout the body (71, 72). Cutaneous mastocytosis, which primarily affects the skin, is more common in children. The most common subtype is known as urticaria pigmentosa, which is characterized by the presence of reddish-brown patches or raised lesions on the skin (Figure 9). When rubbed or irritated, these skin lesions can become itchy and cause hives or flushing (Darrier sign) (73). Most patients have maculopapular lesions. Other common forms of rash are plaque and nodular. Diffuse cutaneous mastocytosis also presents with spontaneous urticaria plaques, not only on the itchy area but also on normal-appearing skin, due to spontaneous or triggered mast cell degranulation. High tryptase levels are measured in mastocytosis, particularly in the presence of widespread multiple or large lesions (72). It can be asymptomatic or sometimes itchy. The systemic forms are associated with flushing and occasionally with wheezing, abdominal pain, diarrhea, and syncope (74).

Systemic mastocytosis is a form that involves the proliferation of mast cells in various organs and tissues throughout the body, including the bone marrow, gastrointestinal tract, liver, spleen, and lymph nodes. Symptoms can vary widely and may include flushing, itching, abdominal pain, diarrhea, low blood pressure, and symptoms associated with allergic reactions (74).

The diagnosis of mastocytosis involves a combination of clinical evaluation, physical examination, skin biopsies, and laboratory tests. Specialized testing, such as bone marrow biopsy and genetic analysis, may be required to assess the extent and subtype of the condition (75).

### 3.7. Urticarial vasculitis

Urticarial vasculitis is a rare condition that combines both urticaria and vasculitis. It is characterized by recurrent episodes of urticaria lasting longer than 24 h which leave behind purpura, a bruise-like discoloration (76, 77). The condition primarily affects the skin, but in some cases, it can involve internal organs and may be a sign of autoimmune disease such as systemic lupus erythetamatosis. The hives in urticarial vasculitis tend to be more persistent, often last for several days or weeks, and can be painful or uncomfortable. They may also be resistant to usual treatments, ie antihistamines, for acute urticaria (78). The hives in urticarial vasculitis may be associated with redness, swelling, and itching. As the hives resolve, there can be remaining purplish or brownish discoloration or induration that can persist for days or weeks (79). Diagnosing urticarial vasculitis involves a combination of clinical evaluation, physical examination, skin biopsies, and laboratory tests such as C3, C4, and C1q complements. Skin biopsy can reveal signs of vasculitis and confirm the diagnosis (80, 81).

### 3.8. Autoinflammatory urticarial syndromes

Autoinflammatory urticarial syndromes, also known as “autoinflammatory diseases with urticarial rash”, constitute a group of rare genetic disorders characterized by recurrent episodes of urticarial rashes accompanied by systemic inflammation. These syndromes are classified within the broader category of autoinflammatory diseases, distinct from autoimmune diseases due to dysregulated innate immune responses instead of abnormal adaptive immune responses.

The hallmark of these syndromes is urticarial rashes presenting as red, itchy, raised patches on the skin with transient presence from hours to days. Lesions usually last up to 24 h and appear as flat erythematous swellings, mostly distributed on the trunk and/or extremities and which do not respond to H1- antihistamines. Often the lesions may be painful and may not be accompanied by itching.

Patients with autoinflammatory urticarial syndromes experience not only skin involvement but also episodes of systemic inflammation. This inflammation can affect various organs and tissues, leading to symptoms such as fever, joint pain, swelling, and occasionally, organ-specific manifestations such as abdominal or chest pain (82).

The primary cause of Autoinflammatory Urticarial Syndromes lies in genetic mutations impacting innate immune system proteins that regulate inflammation and immune responses. The genetic basis varies depending on the specific syndrome, with several genes implicated in different cases (83).

While certain triggers, such as infections, stress, or environmental factors, may exacerbate or provoke rash and inflammation episodes in some cases, they are not universally necessary for symptom development.

The age of onset and duration of symptoms can vary depending on the specific syndrome, with some presenting early in infancy or childhood, while others develop later in life (83).

Autoinflammatory urticarial syndromes encompass different diseases, each with a distinct genetic basis, clinical features, and management considerations.

#### 3.8.1. Cryopyrin-associated periodic syndrome

Cryopyrin-Associated Periodic Syndrome (CAPS) is a rare autoinflammatory disorde, which belongs to a group of conditions known as hereditary periodic fevers. It is caused by mutations in the NLRP3 gene encoding cryopirin, which leads to the overproduction of interleukin-1 beta (IL-1β). There are three subtypes of CAPS, which vary in severity (84).

Familial Cold Autoinflammatory Syndrome (FCAS) is the mildest form of CAPS and is characterized by recurrent episodes of rash, fever, joint pain, and malaise triggered by exposure to cold temperatures. The symptoms typically resolve spontaneously within 24 to 48 h (85).

Muckle-Wells Syndrome (MWS) is an intermediate form of CAPS characterized by recurrent episodes of rash, fever, joint pain, conjunctivitis, and hearing loss. In addition, patients may experience systemic symptoms such as headache, abdominal pain, and fatigue. The symptoms usually last longer than those of FCAS (86).

Neonatal-Onset Multisystem Inflammatory Disease (NOMID) or Chronic Infantile Neurological, Cutaneous, Articular Syndrome (CINCA) is the most severe form of CAPS. It typically presents in infancy and is characterized by persistent inflammation affecting multiple organs, including the skin, joints, eyes, and central nervous system. Patients may experience chronic rash, arthritis, meningitis, hearing loss, developmental delays, and other neurological abnormalities (87).

**Figure 10:**
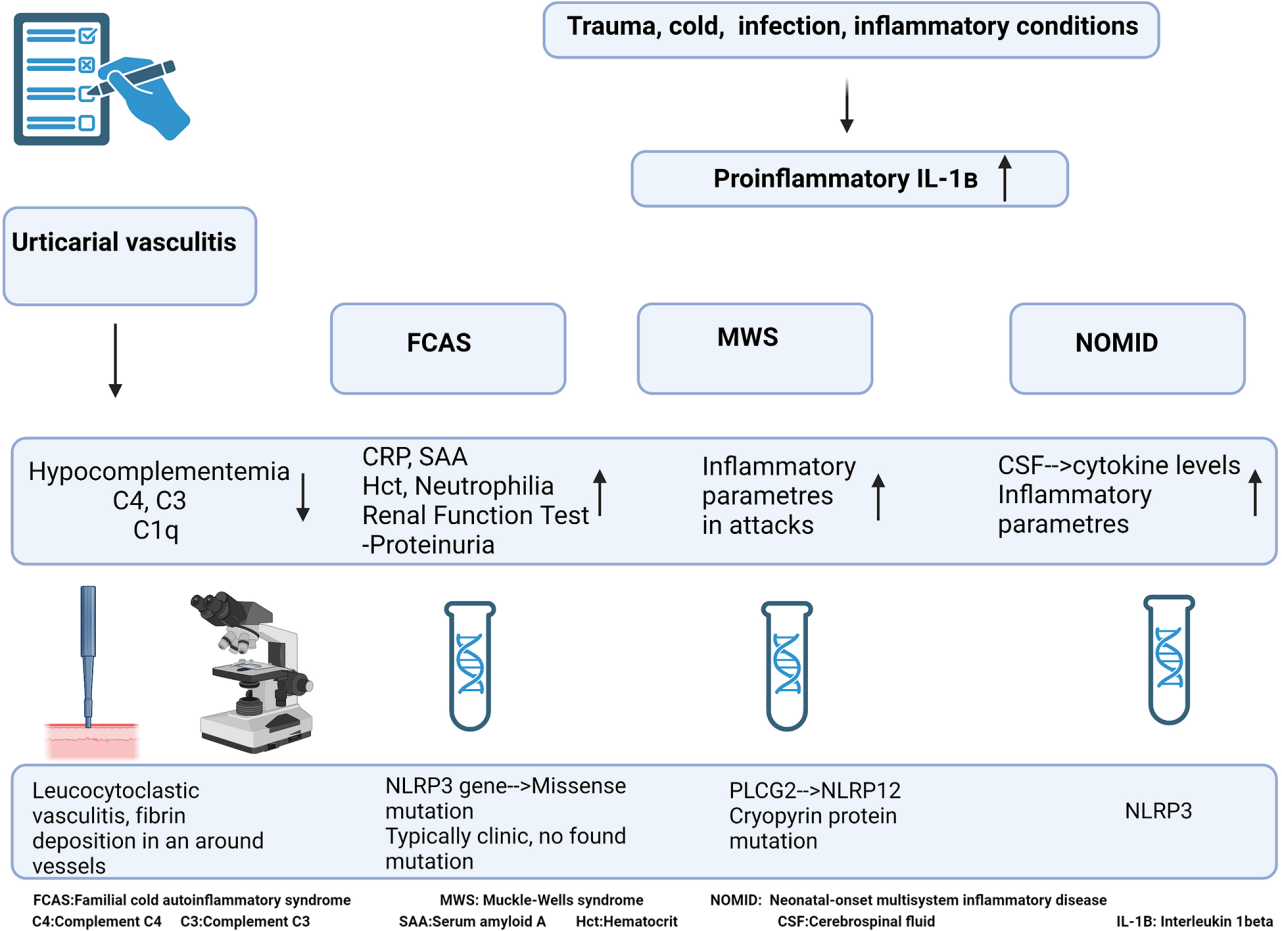
Diagnostic algorithm of cryopyrin-associated periodic syndrome.

When the clinical presentation shows the early onset of recurrent urticaria without itching, triggered by cold, fever, constant musculoskeletal pain, fatigue, and listlessness, CAPS should be considered in the differential diagnosis (88). The diagnosis of CAPS is based on clinical presentation, family history, and genetic testing to identify mutations in the NLRP3 gene, although confirmed NLRP3 gene mutation can be demonstrated only in 40% of individuals affected by CAPS (88). A dramatic response to IL-1β neutralizing drugs is usually observed in treatment. The diagnostic algorithm of CAPS is summarised in Figure 10.

#### 3.8.2. TNF receptor-associated periodic syndrome (TRAPS)

TNF Receptor-Associated Periodic Syndrome is a rare autoinflammatory disorder characterized by recurrent inflammation episodes affecting various body parts (89). It is caused by mutations in the TNFRSF1A gene, which provides instructions for the production of tumor necrosis factor receptor 1 (TNFR1). Although the average age of onset of TRAPS is 4–5 years old, it may not be diagnosed until young adulthood (90).

Individuals with TRAPS experience recurrent episodes of fever that last from 1 to 3 weeks. The fever is often accompanied by fatigue, malaise, and increased levels of acute-phase reactants. TRAPS can cause various cutaneous symptoms, including rash, hyperemia, edema, and urticaria (91). These skin manifestations may occur together with fever or independently. Many people develop severe abdominal pain during the episodes, which can mimic surgical emergencies. Abdominal pain is usually diffuse and can be associated with vomiting (92). TRAPS can cause myalgia, arthralgia, and arthritis, often affecting large joints such as the knees and ankles.

In some cases, TRAPS can involve other organs, such as the eyes, heart, and lungs. Uveitis is a common complication and can lead to permanent vision problems (93). Diagnosing TRAPS involves a combination of clinical evaluation, genetic testing to identify mutations in the TNFRSF1A gene, and ruling out other potential causes of periodic fevers.

Treatment for TRAPS focuses on managing acute episodes and preventing long-term complications. Non-steroidal anti-inflammatory drugs (NSAIDs) and corticosteroids may be used to control symptoms during episodes. Biological medications targeting tumor necrosis factor or interleukin-1 may be considered for individuals with severe or refractory symptoms (94).

#### 3.8.3. Mevalonate kinase deficiency (hyper IgD syndrome)

Mevalonate Kinase Deficiency, Hyper IgD syndrome, is a rare autosomal recessive metabolic disease. It is due to mutations in the MVK gene, which leads to a decrease in the activity of the enzyme mevalonate kinase preventing the conversion of mevalonic acid to phosphomevalonic acid. This results in the accumulation of mevalonic acid and a deficiency in isoprenoid end products. All these changes cause recurrent inflammation (95).

Individuals with Mevalonate Kinase Deficiency experience recurrent episodes of high fever that typically last 3 to 7 days. These febrile episodes can occur at irregular intervals, ranging from weeks to months. The fever episodes often start in early childhood, and together with fever, patients may experience various other symptoms during episodes, such as abdominal pain, diarrhea, arthritis/arthralgia, headache, lymph node enlargement, and skin rash. Cutaneous manifestations include maculopapular rashes, vasculitis, oral aphthous ulcers, and occasionally erythema nodosum (96). Macrophage activation syndrome and AA-amyloidosis are the most severe complications in untreated patients. Different spectrums of diseases are seen in mevalonate kinase deficiency. The rarest and most severe phenotype is mevalonic aciduria (MA), while the inflammatory phenotype is named hyperimmunoglobulin-D periodic fever syndrome (HIDS) due to high serum immunoglobulin D levels in some patients (97). Neurological symptoms may occur in some cases during or between episodes. These include irritability, seizures, ataxia, and developmental delays.

The diagnosis of Mevalonate Kinase Deficiency is based on the combination of clinical evaluation, genetic testing to identify mutations in the MVK gene, and laboratory tests to assess elevated IgD levels during fever episodes.

Treatment focuses on managing the symptoms and preventing complications. Medications such as non-steroidal anti-inflammatory drugs (NSAIDs), corticosteroids, and anti-interleukin-1 (IL-1) neutralizing agents may control inflammation during episodes.

#### 3.8.4. Periodic fever, aphthous stomatitis, pharyngitis, and cervical adenitis syndrome (PFAPA)

Periodic fever, Aphthous stomatitis, Pharyngitis and Cervical Adenitis syndrome is a rare autoinflammatory disorder primarily affecting children. It is characterized by recurrent episodes of high fever (39°C) accompanied by aphthous stomatitis, pharyngitis, and cervical lymphadenopathy (98). These patients experience regular episodes of high fever that typically last for 3 to 6 days. The fever episodes recur at regular intervals of 1 to 2 months in most cases (99). The fever is usually abrupt in onset and may be associated with other symptoms. Aphthous stomatitis commonly occurs during fever episodes and is usually painful. Enlarged lymph nodes, specifically in the cervical region, are a characteristic feature of PFAPA syndrome and can persist beyond the episode. In addition to headache and abdominal pain, splenomegaly may also occur. Although cutaneous manifestations are not common, accompanying rashes are generally in the form of erythema on the trunk, palmoplantar macula, or purpura. Rashes typically develop during fever episodes and can vary in appearance, making it challenging to provide specific characterization. In some cases, they may involve raised, itchy, and transient welts on the skin, which can resemble urticaria.

Aphthae can be seen in the oral and genital mucosa. PFAPA can also be confused with Behçet's disease (99).

The exact cause of PFAPA syndrome is unknown, but it is believed to involve immune system dysregulation. Diagnosis is typically based on the pattern of recurrent fever episodes and the presence of associated symptoms. Laboratory tests are usually performed to rule out other causes of periodic fever syndromes. Most children with PFAPA syndrome eventually outgrow the condition by adolescence.

#### 3.8.5. Schnitzler syndrome

Schnitzler Syndrome is a rare autoinflammatory disorder, and the typical age range of patients with this condition is between 40 and 70 years old. It is more commonly diagnosed in middle-aged to older adults, although cases outside this age range have also been reported, albeit infrequently (100).

Schnitzler syndrome is characterized by the presence of recurrent fever, chronic urticarial, skeletal and muscular pain, fatigue, and monoclonal gammopathy (100). Individuals with Schnitzler syndrome experience periodic fever episodes that can last from days to weeks. Other symptoms often accompany the fever. Chronic urticaria is a hallmark symptom of Schnitzler syndrome, and this may be itchy and typically recur or persist for longer than 6 weeks (101). There may also be associated angioedema. Bone pain is a common feature of Schnitzler syndrome, typically affecting the long bones, and it can be severe and debilitating. Other associated symptoms may include arthralgia, fatigue, weight loss, lymphadenopathy, and organomegaly (79).

The exact cause of Schnitzler syndrome has not been clarified as yet, but it is considered to be an acquired disorder.

Diagnosing Schnitzler syndrome requires a combination of clinical evaluation, the presence of specific symptoms, and laboratory investigations to confirm the presence of monoclonal gammopathy. Laboratory examinations include measurement of levels of acute phase reactants, monoclonal IgM/IgG, serum amyloid A (SAA), S100, A8, A12, serum immune fixation, and skin biopsy (100). Urticarial rash in these patients may not respond to antihistamines and may benefit from corticosteroids. AA-type amyloidosis or lymphoproliferative disease may occur in 15%–20% of untreated patients (101).

#### 3.8.6. Adult onset still's disease (AOSD)

Adult-onset Still's Disease is a rare systemic autoinflammatory disease that is difficult to diagnose. The onset of the disease usually occurs between the ages of 16–82 years (102). Environmental triggers on a genetic background can cause the development of autoinflammatory reactions. Proinflammatory IL-1β plays a role in the pathogenesis of AOSD. The activation of cells such as macrophages and neutrophils can lead to reactive hemophagocytic lymphohistiocytosis (103). Most commonly, Yamaguchi diagnostic criteria are used (104). The classic triad presentation is periodic high fever, arthralgia/arthritis, and a volatile, salmon pink, maculopapular rash (104). The rash is typically evanescent, and commonly described as salmon-pink in colour. While it primarily affects the trunk and limbs, it can also appear on other areas of the body. The rash tends to present symmetrically and may take the form of small macules and papules, resembling urticaria. These skin lesions can vary in size and distribution. Some patients may also experience pruritus. In addition to the salmon-pink rash, AOSD may manifest with erythema nodosum and purpura.

Hepatosplenomegaly and lymphadenopathy are common physical examination findings (102). The diagnostic process includes the determination of CRP, ferritin, and liver function tests. Skin biopsy is particularly important in the case of urticaria since atypical skin findings have been described in 11 of 81 cases in the literature (105, 106).

### 3.9. Wells syndrome and hyper-eosinophilic syndrome

Wells syndrome, also known as eosinophilic cellulitis, eosinophilic dermatitis, or hyper-eosinophilic syndrome, is a rare skin disorder characterized by recurrent episodes of skin inflammation with prominent eosinophilic infiltration (107). The episodes can last for several days to weeks and may recur over months or years. There may be symptom-free intervals between the episodes. Skin lesions are typically red, swollen, and itchy (108). The disease may present with rashes varying from permanent maculopapular rashes to long-lasting lesions in the form of plaque. Skin involvement is present in 50% of patients with HES, and the lesions are accompanied by itching. There are cases of erythematous papules, nodules, urticaria-like lesions, and even angioedema (107). The lesions can vary in size and shape and may tend to migrate or spread. Biopsy of the skin lesions shows a characteristic feature of eosinophil-rich inflammation. Some individuals with Wells syndrome may experience systemic symptoms such as fever, malaise, and eosinophilia (108).

After excluding causes of hypereosinophilia, further investigations should be performed regarding organ involvement, and genetic analysis can clarify the diagnosis (108).

### 3.10. Bullous pemphigoid

**Figure 11:**
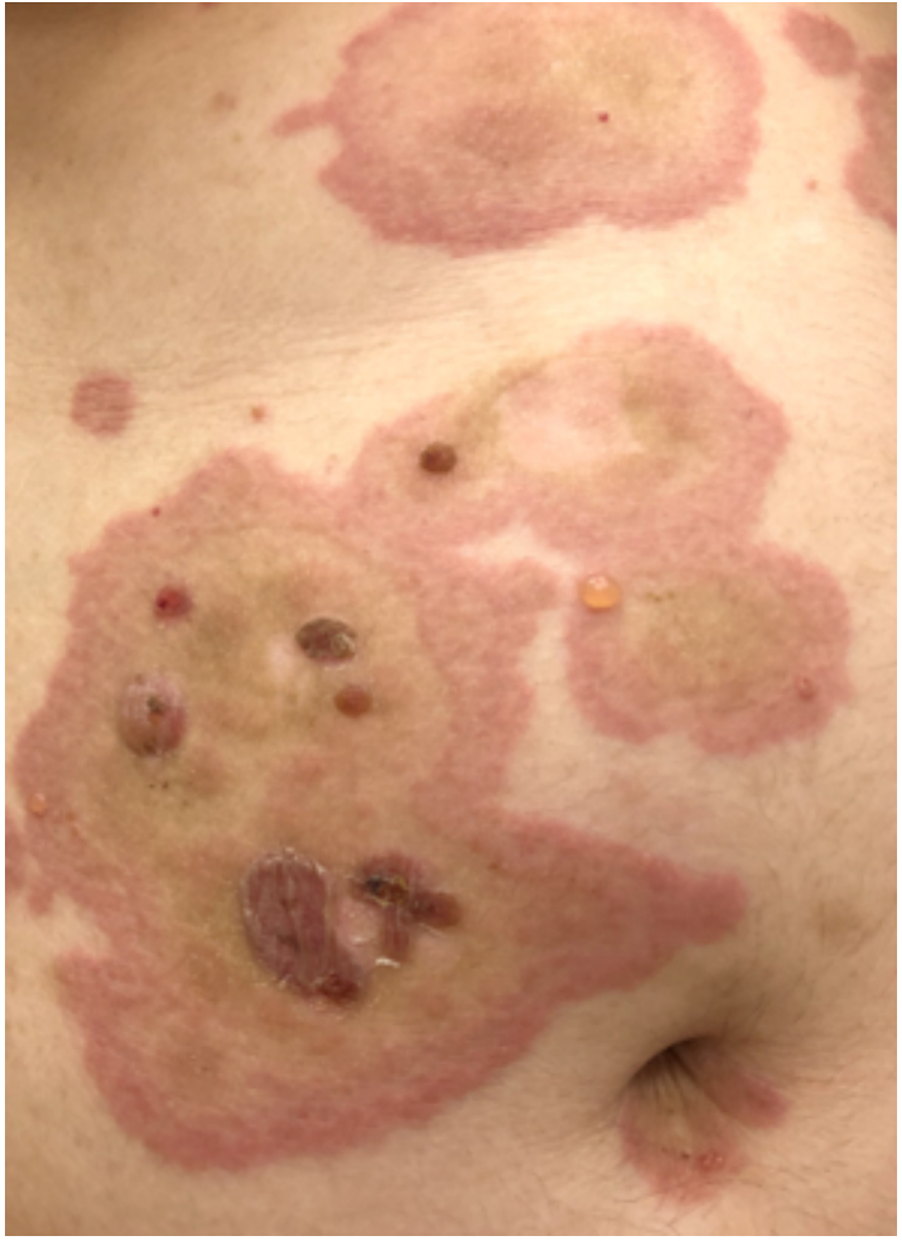
Bullous pemphigoid. Note the small vesicles appearing on the annular urticarial plaques.

Bullous pemphigoid is a chronic autoimmune blistering disorder that primarily affects the elderly, but it has been reported in a wide range of age groups. It generally starts with a non-specific itchy rash and may develop an urticarial appearance over time (109) (Figure 11). Lesions persist for days and may progress to bullae. They can be itchy or painful and may also occur on mucosal surfaces. Bullous pemphigoid is typically a chronic condition with periods of remission and flare-ups. The disease can persist for months to years.

### 3.11. Frey syndrome

Frey syndrome, also known as auriculotemporal syndrome, is a condition characterized by abnormal flushing of the skin on the face, typically triggered by eating certain foods (110). Frey syndrome is commonly seen during infancy or after surgical procedures/trauma involving the parotid gland. It occurs due to nerve damage or misdirection of nerve fibres, leading to abnormal connections between the auriculotemporal nerves (111). The syndrome manifests as the emergence of erythema (mimicking urticaria) and generally on the forehead region within the first few minutes of suckling/eating. During infancy, it can recur in feeding periods and is thought to be due to perinatal trauma of the auriculotemporal nerve. These patients may be misdiagnosed with food allergies, leading to unnecessary diagnostic tests and food eliminations (111).

## 4. Conclusion

Urticaria in childhood is a condition that impairs the quality of life of parents and children and can cause serious anxiety when it appears, does not improve, continues, or recurs. Since urticaria plaque is both the result of many different conditions and can mimic different skin lesions, a wide range of diseases should always be considered in the differential diagnosis. The range and extent of the diseases to be considered in the differential diagnosis can be confusing. A pragmatic approach relies on clinical foresight and knowledge of different clinical situations and their mimics, so that often a simple diagnostic approach can be performed in children without the need for extensive diagnostic work-up.
